# The Relationship between Complement Components *C1R* and *C5* Gene Polymorphism and the Values of Blood Indices in Suckling Piglets

**DOI:** 10.3390/genes14112015

**Published:** 2023-10-28

**Authors:** Hanna Szymańska, Ewa Dzika, Tadeusz Jarosław Zabolewicz, Krystyna Życzko

**Affiliations:** 1 Department of Medical Biology, School of Public Health, Collegium Medicum, University of Warmia and Mazury in Olsztyn, Żołnierska 14C, 10-561 Olsztyn, Poland; 2 Department of Animal Genetics, Faculty of Animal Bioengineering, University of Warmia and Mazury in Olsztyn, Oczapowskiego 5, 10-719 Olsztyn, Poland

**Keywords:** *C1R*, *C5*, complement components, gene polymorphism, blood indices, piglets

## Abstract

The main mechanism of innate immunity is the complement system. Its components include the protein products of the *C1R* and *C5* genes, which are involved in the classical activation pathway as well as the inflammatory and cytolytic immune responses, respectively. The aim of this study was to determine the relationship between PCR-restriction fragment length polymorphism in *C1R* (726T > C) and C5 (1044A > C) genes, and the values of hematological and biochemical blood indices in suckling crossbred (Polish Large White × Polish Landrace × Duroc × Pietrain) piglets (n = 473), considering their age (younger, 21 ± 3 days, n = 274; older, 35 ± 3 days, n = 199) and health status. The frequencies of the *C5* genotypes deviated from the Hardy–Weinberg expectations. Younger piglets, healthy piglets, piglets that deviated from physiological norms and older piglets with the *C1R* TT genotype all had lower white and red blood cell indices. In piglets with the *C5* CC genotype, younger piglets, piglets that deviated from physiological norms and older piglets, a greater number and/or percentage of monocytes were recorded in the blood. Older piglets also showed an increase in the number of leukocytes and granulocytes, along with a tendency for a decrease in the percentage of lymphocytes in their blood. We concluded that a polymorphism in the *C1R* gene may exhibit a functional association or genetic linkage with other genes involved in the process of erythropoiesis. Furthermore the relationship between the *C5* gene polymorphism and the number and/or percentage of monocytes in the blood may modify the body’s defense abilities. Piglets with the CC genotype, having an increased number/proportion of these cells in their blood, probably display a weakened immune response to pathogens or a chronic stimulation of the immune system.

## 1. Introduction

The complement system is the main mechanism of innate immunity. The proteins of this system eliminate the invading pathogens by inducing cell lysis or by stimulating humoral and cellular defense mechanisms [1]. The complement C1r subcomponent, encoded by the *C1R* gene, is a subunit of the C1 complex, which is the first complement component in the classical complement pathway. The C1 complex (790 kDa) consists of one recognition molecule, C1q, and two serine proteases, C1r and C1s, that form a Ca^2+^-dependent C1s-C1r-C1r-C1s heterotetramer. Interactions between C1q and immune complexes lead to C1r autoactivation, which triggers C1s and the downstream substrates C2 and C4 [2]. C1r appears to be important in classical complement activation. A deficiency in C1r function, even in the presence of Ca^2+^ ions, impairs the formation of the C1 complex due to an incorrect association between C1q and C1s components [3]. Moreover, one active C1r subunit, in a mixed dimer with the zymogen form of C1r, was sufficient to fully activate the entire C1 complex [4]. The biosynthesis of C1r was found mainly in hepatocytes, but was also observed in monocytes, endothelial and epithelial cells, myoblasts and chondrocytes [2]. In pigs, C1r was also secreted by aortic smooth muscle cells [5]. In pigs, the *C1R* gene was shown to be involved in the immune response against numerous pathogenic bacteria and viruses [6,7,8,9]. The *C1R* gene was also indicated as being the most important component of the host immune network in the porcine spleen tissue response to *Haemophilus parasuis* infection [10]. In turn, the human *C1R* locus has been found to be highly polymorphic and, thus far, 13 protein alleles have been identified [11]. However, a quantitative analysis of serum C1r has revealed that its deficiency is correlated with the occurrence of autoimmune disorders, such as systemic lupus erythematosus (SLE) and recurrent bacterial infections [2,12]. Polymorphic sites in the *C1R* gene associated with SLE pathogenesis [12,13] and with Periodontal Ehlers-Danlos Syndrome have recently been described [14].

Complement component C5, encoded by the *C5* gene, generates an inflammatory and cytolytic immune response. It is a glycoprotein (196 kDa) mainly synthesized in hepatocytes, monocytes and lymphocytes as a 1976 amino acid precursor, containing a signal peptide and an arginine-rich linker (RPRR) connecting the α and β chains [1]. Acute or chronic inflammation increases the concentration of C5 protein in blood; therefore, it can be considered an acute phase protein (APP) in humans and mice. The activation of C5 by C5 convertases of classical, alternative or lectin pathways leads to the generation of C5a and C5b peptides [15]. Anaphylatoxin C5a induces smooth muscle contractions, increases vascular permeability, mast cell and basophil degranulation, and a phagocyte respiratory burst. As a chemoattractant, C5a is involved in lymphocyte recruitment to the site of infection and directly stimulates the production of cytokines, chemokines and adhesion molecules in immune cells [16,17]. On the bacterial surface, C5b sequentially binds C6, C7, C8 and C9, leading to the formation of a membrane attack complex (C5b-9, MAC), which is responsible for cell lysis and death. Sublytic C5b-9 has a pro-inflammatory effect, including the stimulation of the expression of adhesion molecules and inflammatory mediators in endothelial cells, the synthesis of thromboxane in macrophages and platelets, and the release of reactive oxygen species (ROS) by granulocytes and monocytes [18,19]. The importance of C5 and/or C5b-9 proteins in the immune response has been confirmed in mouse models of pneumococcal meningitidis [16], viral respiratory infection [20] and leptospirosis [21]. In pigs infected with *Actinobacillus pleuropneumonia*, the reduced expression of the *C5* gene in lung tissue has been observed [22]. However, in humans, serum C5 deficiency predisposed subjects to recurrent *Neisseria* sp. infections as well as SLE, otitis media and pneumonia [1,23]. To date, 17 mutations in the human *C5* gene have been identified as causing a serum C5 deficiency [24]. Furthermore, some of the *C5* polymorphic variants were associated with an increased susceptibility to rheumatoid arthritis (RA) [25], SLE, age macular degeneration [26] and other diseases [16,23].

The literature on porcine genes encoding the complement components has focused mainly on studies of the molecular structures of *C3*, *C6*, *C7*, *C8* and *C9* genes, and the association of their single nucleotide polymorphisms (SNPs) with complement activity [27,28,29,30,31,32,33]. In contrast, data concerning the *C1R* and *C5* genes in pigs have been limited to a few publications [34,35]. The *C1R* locus was located on chromosome 5 at position 106 cM, while the *C5* locus, linked to the SW1301 microsatellite, was assigned to chromosome 1q2.13. Ponsuksili et al. [34] identified a SNP 726T > C (GenBank AY349421) in the *C1R* gene, and found comparable *C1R*^T^ and *C1R*^C^ allelic frequencies in Landrace, Large White and Pietrain pig breeds. In turn, Kumar et al. [35] stated that the *C5* gene cDNA comprises 5422 nucleotides (GeneBank AY332748) encoding a 1677-amino-acid protein and detected four SNPs. Only one, at position 1044A > C, differentiated the classical and alternative complement activities and APP levels—C3 and haptoglobin. The authors, based on protein function and association analyses, suggested considering these genes as candidates for innate immunity in pigs.

The known role of *C1R* and *C5* genes may be of particular importance in relation to suckling piglets. At a critical point, between 14 and 35 days after birth, many factors affecting their health and fitness coincide [36]. A four- to five-fold increase in body mass in the 3rd week of life, with accompanying insufficient erythropoiesis, promotes physiological anemia [37]. During this period, passive immunity expires due to the disappearance of colostral antibodies [36]. High growth intensity can also lead to the excessive generation of ROS in their bodies, resulting in oxidative stress and tissue hypoxia [38]. This condition makes the suckling piglets highly susceptible to pathogens. Blood parameters, on the other hand, are useful markers of health status [39]. Therefore, the aim of our study was to determine the relationship between C1R and C5 gene polymorphisms, and the value of hematological and biochemical indices in suckling piglets, considering their age and health status.

## 2. Materials and Methods

### 2.1. Animals and Sample Collection

Blood samples were collected via the jugular vein from 473 suckling, crossbred Polish Large White × Polish Landrace × Duroc × Pietrain piglets that had no visible symptoms of disease and were kept on a private farm in the Warmian–Masurian Voivodeship in Poland. They were divided into younger piglets (n = 274, aged 21 ± 3 days) and older piglets (n = 199, aged 35 ± 3 days). In the first week of life, they received Suibiofer SE (iron preparation) (Biowet Drwalew S.A., Poland), their tails and teeth were removed, and the boars were castrated. Solid feed was introduced from the second week of life. Blood samples were used for molecular, hematological and biochemical examinations. This study was approved by the Local Ethics Committee of the University of Warmia and Mazury in Olsztyn, Poland (Permission Number: 9/2008N). All methods were performed in accordance with the relevant guidelines and regulations.

### 2.2. Molecular Studies

DNA was extracted from peripheral blood leukocytes using the MasterPure™ DNA Purification Kit (EpiCentre, Madison, WI, USA) and its quality was evaluated with a GeneQuant spectrophotometer (Pharmacia LKB Biochrom Ltd., Cambridge, UK). The polymerase chain reaction–restriction fragment length polymorphism (PCR-RFLP) method was used to detect SNPs in *C1R* (726T > C, GenBank AY349421) and *C5* (1044A > C, GeneBank AY332748) genes, with *EcoRV* and *RsaI* restriction enzymes (Fermentas, Lithuania), respectively, as described by Ponsuksili et al. [34], but with some alterations adapted by the Department of Animal Genetics. Each 25 μL PCR reaction contained PCR buffer (20×), 25 mM MgCl2, PCR Enhancer (10×), 2.5 mM dNTPs, 50 μM of each primer, 1 U Tfl DNA polymerase (Epicentre, Madison, WI, USA), approximately 100 ng genomic DNA and deionized, autoclaved water, and was processed in a Thermal Cycler PTC-200 (Bio-Rad, Hercules, CA, USA) in accordance with the following protocol: 94 °C for 5 min., 45 cycles at 94 °C for 30 s; for the *C1R* gene, touchdown annealing temperature from 65 to 60 °C (−0.5 °C/cycle) for 30 s and 35 cycles at 60 °C for 30 s; and for the *C5* gene, 53.3 °C for 30 s, 72 °C for 30 s and a final extension at 72 °C for 5 min. A total of 8 μL of PCR product was incubated with the appropriate restriction enzyme (1 U/μL) at 37 °C for 10–15 min. Restriction fragments were separated by 2.5%–3.5% agarose gel electrophoresis and visualized on a Fluor-S MultiImager (Bio-Rad, Hercules, CA, USA).

### 2.3. Hematological and Biochemical Studies

Hematological and biochemical evaluations were conducted in the Veterinary Diagnostic Laboratory Vetlab Group (Gietrzwałd, Poland). Leukocyte count (WBC), lymphocyte count (LYM) and percentage (%LYM), granulocyte count (GRA) and percentage (%GRA), monocyte count (MONO) and percentage (%MONO), erythrocyte count (RBC), hemoglobin concentration (HGB), mean corpuscular volume (MCV), hematocrit (HCT), mean corpuscular hemoglobin (MCH), mean corpuscular hemoglobin concentration (MCHC), red cell distribution width (RDW), thrombocyte count (PLT), mean platelet volume (MPV), platelet distribution width (PDW), and percentage of large platelets (LPLT) were estimated using an automatic NS4 blood analyzer (Roche Diagnostics, Meylan Cedex, France). High-density lipoprotein cholesterol (HDL-ch), iron (Fe) (BioSystems SA, Barcelona, Spain) and total iron-binding capacity (TIBC) (SPINREACT SA, Barcelona, Spain) were determined according to manufacturer protocol. Transferrin saturation (%Tf), measured as a percentage, was calculated by dividing serum iron concentration by TIBC. Analysis of the values of hematological indices in younger piglets (n = 221) made it necessary to divide them into 3 subgroups by health status: healthy piglets (n = 133, values of blood indices within the reference ranges according to Egeli et al. [37]), anemic piglets (n = 60, hemoglobin level below 8 g/dL and blood values typical of anemia [37]) and piglets that deviated from physiological norms (n = 28, WBC count below and above 11–22 × 10^9^/L, and counts and percentages of leukocyte subpopulations also outside the reference range [37,40,41]). Genotyping and blood chemistry were performed in all 473 piglets (younger, n = 274; older, n = 199). Hematology tests were carried out on 402 piglets (younger, n = 221; older, n = 181); there were 71 blood samples (53 samples from younger piglets and 18 samples from older piglets) where hemolysis occurred.

### 2.4. Statistical Analysis

Characterization of the population genetic structure was made by analyzing allele and genotype frequencies at the locus of the studied genes. The chi-square statistic (χ^2^) was used for testing the Hardy–Weinberg equilibrium and the independence between piglet genotypes and their health status. The Lilliefors-corrected Kolmogorov–Smirnov test was applied to examine if trait values were normally distributed. Non-normal distribution required the use of nonparametric statistics for further investigation. The Mann–Whitney test and the Kruskal–Wallis test with post hoc analysis allowed for the comparison of median values between 2 or 3 groups differing in genotype. Factors such as breed, sex [42,43], relatedness and age had no influence on the values of blood indices in the studied piglets, so they were not included in the statistical analysis. Data were presented in the form of the following descriptive statistics: mean ($\bar{x})$, standard deviation (SD), median (Mdn), minimum (Min) and maximum (Max) values. All calculations were performed with Statistica Version 9.0 (StatSoft Inc., Tulsa, OK, USA).

## 3. Results

### 3.1. Population Structure

In the studied herd of piglets, the SNPs in the *C1R* and *C5* genes were identified (Table 1).

As an effect of *C1R* gene mutation, the genetic equilibrium was maintained, although more heterozygous and less homozygous piglets were observed (Table 1). The deviations between the observed and expected number of individual genotypes were close to the statistically significant value (χ^2^_emp_ = 5.484; χ^2^_tab._ *p* < 0.05 = 5.991). The observed distribution of C5 genotypes deviated significantly from the Hardy–Weinberg equilibrium (χ^2^_emp_ = 63.625; χ^2^_tab._ *p* < 0.01 = 9.210). The reported number of piglets with AA and AC genotypes was lower than assumed. In contrast, the number of CC piglets was greater than expected.

### 3.2. Association Analyses

As presented in Table 2, there was no relationship between the polymorphism of these genes and the health status of younger piglets, and no significant differences were detected between the observed and expected numbers of younger piglets with determined genotypes depending on their health condition.

The results of association studies between *C1R* (726T > C) and *C5* (1044A > C) gene fragment polymorphisms, and the values of hematological and biochemical blood indices in piglets are given in Table 3 and Table 4 as statistically significant differences only. The remaining, not statistically significant results concerning the relationship between SNPs in the studied genes and blood indices in piglets can be found as Supplementary Tables S1–S10 online.

Due to an insufficient number of individuals with the *C1R* CC genotype (n = 4) in the group piglets deviating from physiological norms and *C5* AA in the younger (n = 3), healthy (n = 2) and anemic (n = 1) piglets, it was necessary to combine them into one group with heterozygotes for correct statistical analysis (Table 3 and Table 4, Tables S3, S6, S7 and S9).

Younger piglets, differentiated by *C1R* (726T > C) genotypes, showed a significant difference only in MCHC levels (Table 3).

MCHC values were lower in the blood of TT genotype piglets compared to the TC genotype piglets. In CC piglets, although the level of this indicator was similar to that in heterozygotes, no significant results were observed in relation to either genotype. Healthy piglets, in terms of WBC indices, differed in leukocyte and lymphocyte counts. In heterozygotes, the values of both parameters was higher than in TT homozygotes, while CC piglets with intermediate values did not differ significantly from the above genotypes. Among RBC indices, TT piglets also had the lowest blood concentrations of HGB and the lowest MCHC levels (*p* = 0.058, Table S2), relative to the other genotypic variants, which had levels of these parameters that were almost identical. Likewise, TT piglets that deviated from physiological norms were also characterized by decreased MCHC values compared to CC + TC animals (Table 3). In turn, in anemic piglets, a higher %GRA was noted in the blood of heterozygotes than in opposite homozygotes. Considering older animals, significant results referred to some biochemical, white and red blood cell, and platelet indices as well. The lowest HDL-ch levels were recorded in the CC genotype, distinguishing it from the TC and TT genotypes, which showed nearly the same parameter serum content. TT piglets were found to be associated with the lowest WBC count, including GRA and LYM. The highest values of WBC and GRA were indicated in CC subjects, but they did not differ significantly from heterozygotes in this respect. Differences regarding HGB as well as MCV and MCH levels were also revealed, particularly between TT and TC piglets. TT homozygotes, relative to heterozygotes, had lower HGB, MCH and MCV in the blood. However, CC homozygotes did not differ in this respect from either genotype. HCT levels were also differentiated between the compared genotypes (borderline significance, *p* = 0.054, Table S5). A greater PDW value was found in the blood of older TT piglets compared to TC piglets (Table 3). While CC individuals had PDW parameter values similar to the TC piglets, they did not exhibit any statistical significance with regard to the remaining genotypes.

As shown in Table 4, the *C5* gene polymorphism (1044A > C) was associated with the values of blood indices in younger piglets, including piglets that deviated from physiological norms and anemic piglets, as well as older animals.

Among younger piglets, the CC genotype was recognized as having the highest number and percentage of MONO in the blood out of all WBC parameters. Of note, as CC homozygotes deviated from physiological norms, in relation to heterozygotes, they were also characterized by increased %MONO and RDW values. Likewise, in the healthy and anemic subgroups of CC piglets, such a trend of raised levels and percentages of MONO in blood, although less pronounced (not statistically significant, Tables S7 and S9), was still maintained. Meanwhile, older piglets were found to vary in terms of the levels of biochemical indices, and white and red blood cell indices. In the blood serum of CC individuals, higher TIBC values but lower %Tf were found in comparison to AC piglets. Moreover, this genotype was also distinguished by an elevated WBC count, including MONO and GRA, and a slightly decreased %LYM (borderline significance *p* = 0.055, Table S10). Reduced MCH levels were also noted in CC piglets compared to AC piglets.

To sum up, the *C1R* gene polymorphism (726T > C) (Table 3) indicated lower level of white and red blood cell indices in TT than in CC and TC piglets: younger piglets (MCHC), healthy piglets (WBC, LYM and HGB), piglets that deviated from physiological norms (MCHC) and older piglets (WBC, LYM, GRA, HGB, MCV and MCH). Increased HDL-ch levels and PDW were also noted in older TT piglets. The *C5* gene polymorphism (1044A > C) (Table 4) revealed higher MONO levels and/or %MONO in the CC genotype of younger piglets, piglets that deviated from physiological norms and older piglets. In addition, older CC piglets showed an increase in WBC and GRA, and a trend towards decreased %LYM (Table S10), as well as increased TIBC and decreased %Tf and MCH.

## 4. Discussion

In this study, the *C1R* gene polymorphism revealed similar T and C allele frequencies (Table 1), almost the same as in the Large White pig population (0.55 and 0.45) [34]. However, the authors reported a higher T than C allele frequency in German Landrace (accordingly 0.61 and 0.39) and Pietrain (0.78 and 0.22) pigs. The *C5* gene polymorphism in the studied piglets showed a greater frequency of C alleles than A alleles (Table 1). Ponsuksili et al. [34] also found the C allele more common compared to the A allele in German Landrace (0.97 and 0.03), Large White and Pietrain (0.84 and 0.16) pigs. In our research, a disturbed state of genetic equilibrium was detected (Table 1). Kumar et al. [35] also noted a predominant contribution of the CC genotype in German Landrace (93%) and Pietrain pigs (70%), and a negligible percentage of the AA genotype (0% and 3%, respectively). However, in the populations they studied, the genetic equilibrium was maintained.

Piglets with a TT genotype (*C1R*, 726T > C), whether they were younger, older, healthy, or deviated from physiological norms, showed lower values of white and red blood cell indices (Table 3). In humans, decreased erythrocyte and lymphocyte counts, an increased granulocyte count, and a low HGB concentration were characteristic of iron deficiency [44]. In 21-day-old piglets, iron deficiency did not affect WBC counts or their components, while in 35-day-old piglets, it reduced the number of leukocytes, including granulocytes and eosinophils [37]. The lower number of lymphocytes noted in the blood of TT genotype piglets, according to Özcan et al. [44], may result from the suppression of their production during iron deficiency or their excessive destruction caused by an inefficiency of the antioxidant system. However, our results are difficult to justify with only IDA anemia, because the piglets we studied did not differ in serum TIBC, Fe and %Tf levels. These indicators relatively accurately indicate the state of iron deficiency, even when it is latent [45].

The values of traits in the TT genotype may be explained by the coupling of *C1R* and *IGF-1* loci. The *IGF-1* gene encodes insulin growth factor-1 with pleiotropic action, exerting endocrine and auto/paracrine effects [46]. Like the C1r protein, it is synthesized locally by hepatocytes. Choi and Kim [47] reported that both iron deficiency and anemia were 3–5 times more common in humans with low serum IGF-1 content. Anemia associated with reduced IGF-1 concentrations was attributed to its direct effect on erythroid precursor cell proliferation [48]. In turn, a low amount of blood IGF-1 was also accompanied by a low WBC count, as well as low HGB and Fe levels [47]. A genetic polymorphism within the porcine *IGF-1* gene that affects blood levels was identified [49]. Therefore, the linkage phase of alleles at *C1R* and *IGF-1* loci may play a role in modifying blood cell parameter values. This is obviously just an assumption, because, as is commonly known, IGF-1 levels are influenced not only by gene mutations, but also by other genetic and non-genetic factors [46].

Another assumption concerns the functional link between C1r and IGF-1 proteins. Body fluids and blood contain insulin-like growth factor binding proteins (IGFBP-1-5) that bind IGF-1. These proteins form a complex with IGF-1 and acid labile subunits (ALS), which significantly prolongs the half-life of IGF-1 and its effect on target cells [50]. Moralez et al. [5] indicated that IGFBP-5 proteins control the action of IGF-1 in porcine aortic smooth muscle and human fibroblasts. In these cells, the local production of C1r and C1s proteins was also detected. Interleukin-1 (IL-1) and tumor necrosis factor α (TNF-α) enhanced their secretion, and C1s, triggered by C1r, caused the degradation of IGFBP-5 proteins, leading to a suppression of cellular responses to IGF-1. In dogs with arthritis, active forms of C1r and C1s, along with reduced concentrations of IGFBP-5, IGFBP-3 and IGF-1, were present in their synovial fluid. The inhibition of C1r/C1s activity caused a significant increase in IGFBP-3, IGFBP-5 and IGF-1 levels [51]. Therefore, the mutation of the *C1R* gene may differentiate the force of C1s activation and thus influence the degradation of IGFBP proteins, the cleavage of which modifies the level of IGF-1, which affects erythropoiesis.

In piglets with the CC genotype (*C5*, 1044A > C) that were younger, deviated from physiological norms or were older, a greater number and/or percentage of monocytes were recorded in the blood. Older piglets also showed an increase in the number of leukocytes and granulocytes and a tendency to show a decrease in the percentage of lymphocytes in their blood (Table 4; Table S10).

The first cells attracted to a site of inflammation are neutrophils, followed by (8 h later) monocytes and macrophages [52]. The role of complement in neutrophil recruitment has already been revealed in the bone marrow. During the granulocyte colony-stimulating factor (G-CSF) mobilization of hematopoietic stem/progenitor cells (HSPC), the complement cascade is activated. C5-deficient mice showed an impaired mobilization response to G-CSF and zymosan through the significantly lower number of circulating neutrophils and granulocyte/macrophage progenitors (CFU-GM) found in their blood. This highlights the importance of complement component C5 cleavage fragments (C5a and _desArg_C5a) in granulocyte egress and HSPC mobilization from bone marrow into the bloodstream [53]. In an acute phase reaction, phagocytic neutrophils, monocytes and macrophages secrete proteolytic enzymes, overproduce ROS and release oxygen free radicals [52]. These processes directly activate (non-enzymatically) C5 [54]. Under normal conditions, inflammation is self-limiting and results in healing and complete tissue repair. However, uncontrolled acute inflammation may become chronic [52], which, regardless of the cause, is characterized by increased levels of blood monocytes [55]. These cells were found to be increased in patients with chronic infections, SLE and RA. Susceptibility to such diseases has also been associated with C5 deficiency and/or mutations in the *C5* gene [1].

According to Kumar et al. [35], pigs with the CC genotype (*C5,* 1044A > C) had the lowest classical complement activity and the highest levels of C3c and haptoglobin in their serum. Haptoglobin, as a positive APP, prevents iron loss because of the erythrocyte hemolysis associated with complement activation [52]. Haptoglobin–hemoglobin complexes bind monocyte/macrophage scavenger receptor CD163, which is involved in plasma hemoglobin clearance through receptor-mediated endocytosis. Its soluble form, sCD163, is a marker of low-grade inflammation [56]. Haptoglobin levels are also increased in low-grade inflammation [57].

In addition, Cheng et al. [17] demonstrated the crucial role of C5 in cytokine production by *Candida*-induced mononuclear cells. In human C5-deficient serum, a significant reduction in IL-6, TNF-α and IL-1β production, by 94%, 29% and 75%, respectively, compared to the control serum, was noted. This effect was not observed in sera from C6- or C7-deficient patients. C5 deficiency did not affect the opsonization capacity or phagocytic activity of monocytes. It also had no effect on IFN-γ and IL-17 synthesis by T lymphocytes.

Taking all of this into consideration, an increased number and/or percentage of monocytes in the blood of CC piglets, characteristic of chronic inflammation, may suggest a weaker or delayed activation of their immune defense mechanisms. Perhaps it was the effect of a poor mobilization of immune cells [53] or their impaired synthesis of inflammatory mediators, stimulated by C5 proteins [17]. The lack of correlation between the number of leukocytes and monocytes with indicators of the immune reactivity in CC piglets may confirm this assumption [58].

In this report, we identified for the first time the SNPs in *C1R* (726T > C) and *C5* (1044A > C) genes in Polish commercial crossbred piglets. The conducted association analysis allowed for the indication of genotypes with different levels of immune responsiveness. The results of our research can therefore provide the basis for the genetic improvement of piglet health. This is of measurable importance, as the profitability of pig production is decreased by the costs associated with deaths, mainly of newborns, and diseases of older, suckling piglets. Genetic improvement of the immune potential of suckling piglets would increase the efficiency of their rearing and reduce the expenses associated with the treatment of infectious diseases.

## 5. Conclusions

In the studied population of crossbred piglets, SNPs in *C1R* (726T > C) and *C5* (1044A > C) genes were identified. We concluded that a polymorphism in the *C1R* gene may exhibit a functional association or genetic linkage with other genes involved in the process of erythropoiesis. The relationship between the *C5* gene polymorphism and the number and/or percentage of monocytes in blood may modify the body’s defense abilities. Piglets with the CC genotype, having an increased number/proportion of these cells in their blood, probably display a weakened immune response to pathogens or a chronic stimulation of the immune system.

## Figures and Tables

**Table 1:** The analysis of the genetic population structure.

Mutation	Genotypes	Number and Frequency of Genotypes		Expected Number of Genotypes	χ^2^ Value	*p*-Value	Allele Frequency %	
		n	%				*C1R^C^*	*C1R^T^*
726 T > C	CC	86	18.2	98.79	1.655		45.7	54.3
	TC	260	55.0	234.75	2.716			
	TT	127	26.8	139.46	1.113			
	Together	473	100	473	5.484	0.064		
							*C5* ^A^	*C5^C^*
1044 A > C	AA	3	0.6	27.47	21.798		24.1	75.9
	AC	222	47.0	272.49	9.355			
	CC	248	52.4	173.04	32.472			
	Together	473	100	473	63.625	<0.001		

χ^2^, chi-square value; df 2: χ^2^_.05_ = 5.991, the tabular χ^2^ value at 2 degrees of freedom (df) and a probability (*p*) of 0.05; df 2: χ^2^_.01_ = 9.210, the tabular χ^2^ value at 2 degrees of freedom (df) and a probability (*p*) of 0.01.

**Table 2:** The association between the studied genes polymorphism and the health status of younger piglets.

Younger Piglets	Observed and (Expected) Number of Piglets			Together	Sum of χ^2^	df	*p*-Value
	*C1R* Genotypes						
	CC	TC	TT				
Healthy	26 (27.08)	71 (72.22)	36 (33.70)	133	0.221		
Deviated from physiological norm	4 (5.70)	14 (15.20)	10 (7.10)	28	1.786		
Anemic	15 (12.22)	35 (32.58)	10 (15.20)	60	2.591		
Together	45	120	56	221	4.598	4	0.330
	*C5* genotypes						
	AA	AC	CC				
Healthy	2 (1.81)	71 (73.42)	60 (57.77)	133	0.186		
Deviated from physiological norm	0 (0.38)	15 (15.46)	13 (12.16)	28	0.452		
Anemic	1 (0.81)	36 (33.12)	23 (26.07)	60	0.656		
Together	3	122	96	221	1.294	4	0.863

χ^2^, chi-square value; df 4:χ^2^_.05_ = 9.488, the tabular χ^2^ value at 4 degrees of freedom (df) and a probability (*p*) of 0.05; df 4:χ^2^_.01_ = 13.277, the tabular χ^2^ value at 4 degrees of freedom (df) and a probability (*p*) of 0.01.

**Table 3:** The relationship between *C1R* genotypes and the values of hematological and biochemical indices in suckling piglets.

Piglets	Indices		Genotypes			*p*-Value
Younger			CC, n = 45	TC, n = 120	TT, n = 56	
	MCHC (g/dL)	$\bar{x}\;$± SD	34.17 ± 2.629	34.34 ± 2.501	33.11 ± 2.209	0.010
		Mdn (Min–Max)	34.10 (28.90–40.00)	34.40 ^A^ (28.20–42.70)	32.95 ^B^ (28.20–37.50)	
Healthy			CC, n = 26	TC, n = 71	TT, n = 36	
	WBC (10^9^/L)	$\bar{x}\;$± SD	15.24 ± 2.744	16.13 ± 3.547	13.94 ± 3.290	0.009
		Mdn (Min–Max)	15.31 (9.86–20.98)	16.21 ^A^ (8.41–22.85)	14.10 ^B^ (6,51–21.08)	
	LYM (10^9^/L)	$\bar{x}\;$± SD	6.87 ± 1.673	7.40 ± 1.968	6.29 ± 1.694	0.014
		Mdn (Min–Max)	6.95 (3.40–9.90)	7.60 ^A^ (3.50–12.90)	6.00 ^B^ (3.50–9.90)	
	HGB (g/dL)	$\bar{x\;}$± SD	10.44 ± 1.401	10.14 ± 1.432	9.61 ± 1.047	0.047
		Mdn (Min–Max)	10.05 (8.40–14.40)	10.00 (8.10–15.20)	9.60 (8.00–12.10)	
Deviated from physiological norm			CC + TC, n = 18		TT, n = 10	
	MCHC (g/dl)	$\bar{x}\;$± SD	35.02 ± 1.723		33.07 ± 2.146	0.041
		Mdn (Min–Max)	35.00 (32.10–39.40)		32.70 (29.10–35.50)	
Anemic			CC, n = 15	TC, n = 35	TT, n = 10	
	%GRA (%)	$\bar{x}\;$± SD	32.47 ± 4.441	37.37 ± 6.392	34.93 ± 8.042	0.031
		Mdn (Min–Max)	32.41 ^a^ (24.50–40.47)	36.19 ^b^ (22.39–53.54)	32.64 (24.24–46.88)	
Older			CC, n = 29	TC, n = 113	TT, n = 57	
	HDL-ch (mg/dL)	$\bar{x\;}$± SD	45.45 ± 11.278	51.13 ± 12.265	51.09 ± 10.683	0.022
		Mdn (Min–Max)	44.00 ^a^ (29.00–73.00)	49.00 ^b^ (23.00–83.00)	50.00 ^b^ (20.00–80.00)	
			n = 25	n = 104	n = 52	
	WBC (10^9^/L)	$\bar{x}\;$± SD	22.54 ± 5.157	21.66 ± 6.100	19.15 ± 6.404	0.012
		Mdn (Min–Max)	23.89 ^b^ (11.92–30.11)	20.60 ^b^ (10.93–39.03)	17.92 ^a^ (8.44–37.60)	
	LYM (10^9^/L)	$\bar{x}\;$± SD	8.49 ± 2.039	8.28 ± 2.275	7.48 ± 2.474	0.034
		Mdn (Min–Max)	8.70 (5.50–12.80)	8.00 (4.40–14.90)	6.70 (3.70–16.10)	
	GRA (10^9^/L)	$\bar{x}$ ± SD	12.00 ± 3.543	11.49 ± 4.180	9.84 ± 3.977	0.017
		Mdn (Min–Max)	12.40 ^b^ (5.20–18.80)	11.05 ^b^ (4.20–24.30)	9.05 ^a^ (4.10–19.00)	
	HGB (g/dL)	$\bar{x}\;$± SD Mdn (Min–Max)	11.12 ± 0.855	11.39 ± 1.072	10.84 ± 1.086	0.007
			11.20 (9.30–12.40)	11.40 ^B^ (8.10–14.10)	10.80 ^A^ (8.90–13.70)	
	MCV (fL)	$\bar{x}\;$± SD	49.57 ± 3.522	50.99 ± 4.185	49.15 ± 4.021	0.026
		Mdn (Min–Max)	49.80 (42.20–55.80)	51.00 ^b^ (40.90–59.70)	50.15 ^a^ (41.00–57.60)	
	MCH (p/g)	$\bar{x}\;$± SD	17.00 ± 0.836	17.46 ± 1.295	16.92 ± 0.942	0.013
		Mdn (Min–Max)	16.80 (15.40–18.60)	17.30 ^b^ (15.00–24.10)	16.80 ^a^ (15.30–19.20)	
	PDW (%)	$\bar{x\;}$± SD	7.56 ± 0.840	7.37 ± 1.060	7.81 ± 1.027	0.028
		Mdn (Min–Max)	7.30 (6.30–9.20)	7.15 ^b^ (5.20–9.90)	8.00 ^a^ (5.70–9.40)	

$\bar{x},$ mean; SD, standard deviation; Mdn, median; Min–Max, minimum and maximum value. Medians in rows with different letters differ significantly: lowercase letters a, b at *p* ≤ 0.05; uppercase letters A, B at *p* ≤ 0.01; medians in rows with the same letters (A, a or B, b) do not differ significantly.

**Table 4:** The relationship between *C5* genotypes and the values of hematological and biochemical indices in suckling piglets.

Piglets	Indices		Genotypes:		*p*-Value
Younger			AA + AC, n = 125	CC, n = 96	
	MONO (10^9^/L)	$\bar{x}\;$± SD	1.45 ± 0.563	1.71 ± 0.717	0.001
		Mdn (Min–Max)	1.40 (0.30–3.50)	1.50 (0.50–4.50)	
	%MONO (%)	$\bar{x\;}$± SD	10.08 ± 2.787	10.75 ± 2.860	0.050
		Mdn (Min–Max)	9.23 (6.49–17.96)	9.35 (6.22–16.97)	
Deviated from physiological norm			AC, n = 15	CC, n = 13	
	%MONO (%)	$\bar{x}\;$± SD	8.23 ± 0.981	9.71 ± 2.554	0.030
		Mdn (Min–Max)	8.34 (6.87–9.96)	9.14 (6.97–16.72)	
	RDW (%)	$\bar{x\;}$± SD	15.10 ± 2.577	18.28 ± 3.467	0.010
		Mdn (Min–Max)	15.00 (11.60–19.80)	17.70 (12.70–27.80)	
Older			AC, n = 76	CC, n = 123	
	TIBC (µmol/L)	$\bar{x\;}$± SD	89.86 ± 23.699	96.77 ± 21.620	0.018
		Mdn (Min–Max)	82.21 (36.00–144.00)	97.70 (20.42–150.27)	
	%Tf (%)	$\bar{x\;}$± SD	26.06 ± 7.869	24.09 ± 10.479	0.017
		Mdn (Min–Max)	25.54 (7.76–59.20)	23.52 (6.42–41.36)	
			n = 69	n = 112	
	WBC (10^9^/L)	$\bar{x}\;$± SD	19.29 ± 5.104	22.15 ± 6.523	0.006
		Mdn (Min–Max)	19.24 (8.44–29.10)	21.35 (10.90–39.03)	
	MONO (10^9^/L)	$\bar{x}\;$± SD	1.73 ± 0.562	2.02 ± 0.745	0.011
		Mdn (Min–Max)	1.70 (0.70–3.50)	1.80 (0.90–4.60)	
	GRA (10^9^/L)	$\bar{x\;}$± SD	9.88 ± 3.342	11.84 ± 4.355	0.004
		Mdn (Min–Max)	9.20 (4.10–16.80)	11.65 (4.20–24.30)	
	MCH (pg)	$\bar{x}\;$± SD	17.43 ± 1.051	17.13 ± 1.299	0.031
		Mdn (Min–Max)	17.40 (15.30–19.60)	17.00 (15.00–24.10)	

$\bar{x,}$ mean; SD, standard deviation; Mdn, median; Min–Max, minimum and maximum values.

## Data Availability

All data supporting this study are included in this article and Supplementary Materials, or are available upon request.

## Supplementary Materials

The following supporting information can be downloaded at: https://www.mdpi.com/article/10.3390/genes14112015/s1. Table S1: The relationship between *C1R* genotypes and the values of hematological and biochemical indices in younger piglets. Table S2: The relationship between *C1R* genotypes and the values of hematological and biochemical indices in healthy piglets. Table S3: The relationship between *C1R* genotypes and the values of hematological and biochemical indices in piglets that deviated from physiological norms. Table S4: The relationship between *C1R* genotypes and the values of hematological and biochemical indices in anemic piglets. Table S5: The relationship between *C1R* genotypes and the values of hematological and biochemical indices in older piglets. Table S6: The relationship between *C5* genotypes and the values of hematological and biochemical indices in younger piglets. Table S7: The relationship between *C5* genotypes and the values of hematological and biochemical indices in healthy piglets. Table S8: The relationship between *C5* genotypes and the values of hematological and biochemical indices in piglets that deviated from physiological norms. Table S9: The relationship between *C5* genotypes and the values of hematological and biochemical indices in anemic piglets. Table S10: The relationship between *C5* genotypes and the values of hematological and biochemical indices in older piglets.
