# Supplementary material for: The Relationship between Complement Components C1R and C5 Gene Polymorphism and the Values of Blood Indices in Suckling Piglets
Source: Genes (Basel). 2023 Oct 28;14(11):2015. doi: 10.3390/genes14112015 (PMC10671359; doi:10.3390/genes14112015)
Supplement: Supplementary file 1 [file genes-14-02015-s001.zip › genes-2661077-supplementary.pdf]

The Relationship between Complement Components *C1R* and *C5* Gene Polymorphism and the Values of Blood Indices in Suckling Piglets

Hanna Szymańska [1,*], Ewa Dzika [1], Tadeusz Jarosław Zabolewicz [2], Krystyna Życzko [2]

[1] Department of Medical Biology, School of Public Health, Collegium Medicum, University of Warmia and Mazury in Olsztyn, Żołnierska 14C, 10-561 Olsztyn, Poland.

[2] Department of Animal Genetics, Faculty of Animal Bioengineering, University of Warmia and Mazury in Olsztyn, Oczapowskiego 5, 10-719 Olsztyn, Poland.

* Correspondence: hanna.szymanska@uwm.edu.pl

Table S1. The relationship between *C1R* genotypes and the values of hematological and biochemical indices in younger piglets.

Indices		Genotypes<br>CC, n=57	 TC, n=147	 TT, n=70	*P*-value
HDL-ch (mg/dL)	$\bar{x}$±SD	61.04±13.477	62.85±15.008	61.63±15.191	0.657
	Mdn (Min-Max)	61.00 (31.00-89.00)	62.00 (29.00-112.00)	60.00 (37.00-106.00)	
TIBC (µmol/L)	$\bar{x}$±SD	122.85±27.973	129.93±32.339	121.47±31.920	0.065
	Mdn (Min-Max)	122.77 (50.33-204.00)	130.47 (60.00-216.35)	116.50 (54.63-213.13)	
Fe (µmol/L)	$\bar{x}$±SD	14.94±8.409	13.00±8.215	12.47±6.428	0.121
	Mdn (Min-Max)	12.90 (3.40-51.04)	10.75 (2.69-51.22)	10.57 (3.58-36.54)	
% Tf (%)	$\bar{x}$±SD	12.67±7.531	10.96±7.540	11.17±6.620	0.240
	Mdn (Min-Max)	10.83 (2.62-32.37)	8.26 (1.32-40.74)	9.58 (2.16-32.28)	
		n=45	n=120	n=56	
WBC (10^9/L)	$\bar{x}$±SD	14.40±4.528	15.84±5.547	15.15±5.716	0.207
	Mdn (Min-Max)	14.65 (5.81-26.91)	15.59 (1.67-35,89)	14.10 (6.51-30.64)	
LYM (10^9/L)	$\bar{x}$±SD	6.72±2.212	7.33±2.757	6.78±2.558	0.160
	Mdn (Min-Max)	6.90 (2.70-12.70)	7.20 (0.90-19.10)	6.00 (3.50-17.20)	
%LYM (%)	$\bar{x}$±SD	47.54±9.884	46.96±8.712	45.85±8.345	0.337
	Mdn (Min-Max)	49.70 (26.52-63.68)	46.17 (26.09-64.68)	45.31 (25.51-63.29)	
MONO (10^9/L)	$\bar{x}$±SD	1.61±0.791	1.57±0.619	1.52±0.577	0.851
	Mdn (Min-Max)	1.40 (0.50-4.50)	1.50 (0.30-3.60)	1.40 (0.60-3.00)	
%MONO (%)	$\bar{x}$±SD	10.98±0.062	10.19±2.800	10.27±2.691	0.292
	Mdn (Min-Max)	9.54 (6.64-16.97)	9.16 (6.50-17.96)	9.36 (6.22-16.08)	
GRA (10^9/L)	$\bar{x}$±SD	6.05±2.679	6.95±3.141	6.88±3.433	0.272
	Mdn (Min-Max)	5.60 (1.60-14.80)	6.20 (0.50-15.40)	6.10 (2.10-15.70)	
%GRA (%)	$\bar{x}$±SD	41.33±11.279	42.97±9.242	44.03±9.100	0.189
	Mdn (Min-Max)	39.77 (24.50-65.82)	42.19 (22.39-65.51)	43.40 (24.24-67.88)	
RBC (10^{12}/L)	$\bar{x}$±SD	5.51±1.026	5.45±1.096	5.48±0.987	0.987
	Mdn (Min-Max)	5.73 (3.71-7.64)	5.60 (2.75-7.59)	5.68 (2.25-7.41)	
HGB (g/dL)	$\bar{x}$±SD	9.55±1.763	9.33±1.974	9.26±1.680	0.905
	Mdn (Min-Max)	9.50 (7.10-14.40)	9.35 (4.80-15.20)	9.25 (3.90-12.20)	
MCV (fL)	$\bar{x}$±SD	51.61±5.833	50.22±5.688	51.40±4.806	0.170
	Mdn (Min-Max)	52.90 (40.70-60.80)	49.65 (38.90-64.60)	50.50 (40.50-60.90)	
HCT (%)	$\bar{x}$±SD	28.09±5.605	27.47±5.947	27.96±4.915	0.520
	Mdn (Min-Max)	26.90 (20.00-41.40)	27.20 (12.50-43.30)	28.50 (13.40-37.40)	
MCH (pg)	$\bar{x}$±SD	17.36±1,494	17.11±1.569	16.92±1.488	0.361
	Mdn (Min-Max)	17.40 (14.50-20.60)	16.90 (14.00-21.80)	17.20 (13.60-20.10)	
RDW (%)	$\bar{x}$±SD	17.68±3.293	17.96±3.487	18.05±2.825	0.884
	Mdn (Min-Max)	17.50 (11.70-27.20)	17.85 (11.10-26.00)	17.35 (12.70-27.80)	
PLT (10^9/L)	$\bar{x}$±SD	967.34±455.104	987.55±388.876	1009.68±377.326	0.799
	Mdn (Min-Max)	835.00 (229.00-1838.00)	960.50 (55.00-1842.00)	985.50 (377.00-2286.00)	
MPV (fL)	$\bar{x}$±SD	11.16±0.519	11.09±0.537	11.02±0.464	0.447
	Mdn (Min-Max)	11.10 (10.20-12.40)	11.00 (9.70-12.30)	11.00 (10.10-12.30)	
LPLT (%)	$\bar{x}$±SD	1.07±0.465	1.09±0.411	1.11±0.393	0.853
	Mdn (Min-Max)	1.00 (0.25-1.95)	1.08 (0.06-1.95)	1.09 (0.41-2.40)	
PDW (%)	$\bar{x}$±SD	7.90±0.919	7.82±1.103	7.51±0.984	0.133
	Mdn (Min-Max)	7.90 (6.30-9.50)	7.80 (5.50-12.20)	7.50 (5.50-9.50)	

$\bar{x}$, mean; SD, standard deviation; Mdn, median; Min-Max, minimum and maximum value.

Table S2. The relationship between *C1R* genotypes and the values of hematological and biochemical indices in healthy piglets.

Indices		Genotypes CC, n=26	TC, n=71	TT, n=36	*P*-value
HDL-ch (mg/dL)	$\bar{x}$±SD	62.92±11.544	62.62±13.971	62.22±14.850	0.846
	Mdn (Min-Max)	61.00 (45.00-88.00)	62.00 (30.00-103.00)	60.00 (39.00-106.00)	
TIBC (μmol/L)	$\bar{x}$±SD	125.22±24.390	122.77±29.081	124.20±32.299	0.789
	Mdn (Min-Max)	125.28 (71.82-183.04)	124.30 (64.48-193.79)	120.63 (72.36-198.44)	
Fe (μmol/L)	$\bar{x}$±SD	14.54±6.682	13.87±8.954	11.96±5.797	0.269
	Mdn (Min-Max)	14.15 (6.27-34.21)	11.37 (3.58-51.22)	10.57 (3.58-27.40)	
% Tf (%)	$\bar{x}$±SD	12.51±7.127	12.14±8.923	10.38±5.758	0.480
	Mdn (Min-Max)	12.11 (4.01-32.37)	9.70 (2.29-40.74)	9.58 (2.16-28.51)	
%LYM (%)	$\bar{x}$±SD	45.19±8.395	46.18±8.521	45.25±5.967	0.902
	Mdn (Min-Max)	45.89 (28.22-60.85)	45.40 (28.51-62.88)	45.08 (29.90-57.65)	
MONO (10^9/L)	$\bar{x}$±SD	1.58±0.564	1.53±0.498	1.47±0.568	0.636
	Mdn (Min-Max)	1.45 (0.80-2.70)	1.50 (0.60-2.60)	1.30 (0.60-3.00)	
%MONO (%)	$\bar{x}$±SD	10.23±2.761	9.50±2.325	10.46±2.735	0.130
	Mdn (Min-Max)	9.27 (6.64-16.97)	8.82 (6.50-16.11)	9.44 (6.22-16.08)	
GRA (10^9/L)	$\bar{x}$±SD	6.75±2.000	7.20±2.278	6.19±1.731	0.104
	Mdn (Min-Max)	6.80 (4.00-11.20)	6.70 (2.60-12.70)	6.10 (2.40-12.10)	
%GRA (%)	$\bar{x}$±SD	44.24±10.072	44.41±8.861	44.42±6.568	0.987
	Mdn (Min-Max)	43.28 (24.59-64.73)	44.83 (28.19-63.60)	43.12 (34.16-62.37)	
RBC (10^{12}/L)	$\bar{x}$±SD	6.08±0.671	5.96±0.650	5.74±0.563	0.098
	Mdn (Min-Max)	6.07 (4.79-7.64)	5.95 (4.53-7.37)	5.75 (4.27-6.85)	
MCV (fL)	$\bar{x}$±SD	51.11±5.510	49.93±5.708	51.18±5.109	0.421
	Mdn (Min-Max)	51.70 (40.70-60.10)	49.60 (39.70-62.10)	50.05 (40.50-60.90)	
HCT (%)	$\bar{x}$±SD	30.85±4.907	29.69±4.436	29.18±3.047	0.255
	Mdn (Min-Max)	31.90 (21.90-41.40)	29.70 (20.10-43.30)	29.25 (22.10-35.50)	
MCH (pg)	$\bar{x}$±SD	17.15±1.439	16.96±1.443	16.77±1.612	0.615
	Mdn (Min-Max)	17.30 (14.50-19.80)	16.70 (14.00-20.60)	17.00 (13.60-20.10)	
MCHC (g/dL)	$\bar{x}$±SD	34.08±2.916	34.26±2.726	32.96±2.285	0.058
	Mdn (Min-Max)	34.20 (28.90-39.90)	34.60 (28.20-42.70)	32.85 (28.20-37.40)	
RDW (%)	$\bar{x}$±SD	16.28±2.290	16.55±2.614	17.34±1.807	0.209
	Mdn (Min-Max)	16.55 (11.70-20.20)	16.60 (11.10-23.50)	16.90 (14.80-21.20)	
PLT (10^9/L)	$\bar{x}$±SD	949,77±427.103	1014.37±370.900	998.47±295.178	0.667
	Mdn (Min-Max)	892.50 (327.00-1668.00)	983.00 (211.00-1842.00)	1012.50 (377.00-1662.00)	
MPV (fL)	$\bar{x}$±SD	11.04±0.516	10.97±0.560	10.99±0.397	0.692
	Mdn (Min-Max)	11.10 (10.20-12.40)	10.90 (9.70-12.30)	11.00 (10.10-12.00)	
LPLT (%)	$\bar{x}$±SD	1.04±0.449	1.11±0.389	1.09±0.318	0.672
	Mdn (Min-Max)	0.97 (0.37-1.79)	1.08 (0.23-1.95)	1.12 (0.41-1.78)	
PDW (%)	$\bar{x}$±SD	7.63±0.892	7.56±1.005	7.43±0.835	0.786
	Mdn (Min-Max)	7.30 (6.30-9.20)	7.40 (5.70-9.60)	7.35 (5.50-9.40)	

$\bar{x}$, mean; SD, standard deviation; Mdn, median; Min-Max, minimum and maximum value.

Table S3. The relationship between *C1R* genotypes and the values of hematological and biochemical indices in piglets deviated from physiological norm.

Indices		Genotypes CC+TC, n=18	TT, n=10	*P*-value
HDL-ch	$\bar{x}$±SD	57.00±14.418	54.30±14.515	0.597
(mg/dL)	Mdn (Min-Max)	55.50 (31.00-86.00)	53.00 (37.00-83.00)	
TIBC	$\bar{x}$±SD	130.58±29.932	122.04±24.883	0.549
(µmol/L)	Mdn (Min-Max)	135.85 (87.94-186.25)	116.86 (92.95-173.73)	
Fe	$\bar{x}$±SD	16.42±9.984	17.28±9.155	0.649
(µmol/L)	Mdn (Min-Max)	11.01 (5.19-38.87)	17.37 (5.37-36.54)	
% Tf	$\bar{x}$±SD	14.21±10.160	15.43±9.121	0.755
(%)	Mdn (Min-Max)	8.81 (3.22-31.54)	15.96 (3.09-32.28)	
WBC	$\bar{x}$±SD	23.01±6.487	25.04±2.448	0.981
(10^9/L)	Mdn (Min-Max)	24.89 (10.18-35.89)	24.20 (22.94-30.64)	
LYM	$\bar{x}$±SD	9.46±4.275	10.17±3.339	0.701
(10^9/L)	Mdn (Min-Max)	8.80 (2.70-19.10)	10.75 (5.90-17.20)	
%LYM	$\bar{x}$±SD	39.81±10.277	40.10±10.374	0.981
(%)	Mdn (Min-Max)	40.68 (26.08-55.27)	40.69 (25.51-56.14)	
MONO	$\bar{x}$±SD	2.18±0.950	2.08±0.305	0.942
(10^9/L)	Mdn (Min-Max)	2.10 (0.70-4.50)	2.10 (1.6-2.50)	
%MONO	$\bar{x}$±SD	9.26±2.341	8.31±0.943	0.350
(%)	Mdn (Min-Max)	8.75 (6.88-16.72)	8.58 (6.92-9.47)	
GRA	$\bar{x}$±SD	11.38±3.111	12.82±2.135	0.302
(10^9/L)	Mdn (Min-Max)	11.30 (5.70-15.40)	13.05 (8.20-15.70)	
%GRA	$\bar{x}$±SD	50.98±11.080	51.72±10.718	0.905
(%)	Mdn (Min-Max)	51.01 (34.89-65.82)	49.99 (35.64-67.88)	
RBC	$\bar{x}$±SD	6.29±0.704	5.87±1.408	0.684
(10^{12}/L)	Mdn (Min-Max)	6.53 (4.76-7.59)	5.91 (2.25-7.41)	
HGB	$\bar{x}$±SD	10.75±1.717	10.05±2.533	0.773
(g/dL)	Mdn (Min-Max)	10.95 (8.20-14.30)	10.90 (3.90-12.20)	
MCV	$\bar{x}$±SD	48.94±5.840	51.92±4.277	0.314
(fL)	Mdn (Min-Max)	50.00 (38.90-57.00)	50.55 (44.50-59.60)	
HCT	$\bar{x}$±SD	30.84±5.689	30.09±6.753	0.924
(%)	Mdn (Min-Max)	31.85 (20.80-41.60)	32.05 (13.40-37.40)	
MCH	$\bar{x}$±SD	17.01±1.621	17.08±1.284	0.943
(pg)	Mdn (Min-Max)	16.85 (14.60-19.30)	17.35 (14.10-19.00)	
RDW	$\bar{x}$±SD	16.09±2.878	17.46±4.149	0.517
(%)	Mdn (Min-Max)	16.10 (11.60-20.80)	16.65 (12.70-27.80)	
PLT	$\bar{x}$±SD	788.83±420.115	881.30±543.364	0.792
(10^9/L)	Mdn (Min-Max)	821.00 (55.00-1413.00)	773.50 (385.00-2286.00)	
MPV	$\bar{x}$±SD	11.19±0.404	11.03±0.508	0.718
(fL)	Mdn (Min-Max)	11.20 (10.50-12.00)	11.25 (10.20-11.50)	
LPLT	$\bar{x}$±SD	0.88±0.472	0.96±0.559	0.719
(%)	Mdn (Min-Max)	0.97 (0.06-1.59)	0.85 (0.44-2.40)	
PDW	$\bar{x}$±SD	8.04±1.509	7.44±1.000	0.157
(%)	Mdn (Min-Max)	8.35 (5.50-12.20)	8.00 (5.70-8.30)	

$\bar{x}$, mean; SD, standard deviation; Mdn, median; Min-Max, minimum and maximum value.

Table S4. The relationship between *C1R* genotypes and the values of hematological and biochemical indices in anemic piglets.

Indices		Genotypes CC, n=15	TC, n=35	TT, n=10	*P*-value
HDL-ch	$\bar{x}$±SD	65.40±15.061	62.09±18.204	64.90±14.985	0.624
(mg/dL)	Mdn (Min-Max)	64.00 (31.00-89.00)	62.00 (29.00-106.00)	65.00 (42.00-93.00)	
TIBC	$\bar{x}$±SD	123.60±37.643	141.38±35.153	113.87±24.412	0.057
(μmol/L)	Mdn (Min-Max)	132.18 (50.33-204.00)	142.74 (81.67-208.83)	119.19 (54.63-138.80)	
Fe	$\bar{x}$±SD	11,07±6.306	11.07±5.507	11.36±4.898	0.961
(μmol/L)	Mdn (Min-Max)	10.39 (3.40-25.43)	10.39 (2.69-24.72)	8.78 (6.45-20.06)	
% Tf	$\bar{x}$±SD	9.88±6.348	8.37±4.655	10.38±4.381	0.452
(%)	Mdn (Min-Max)	7.59 (2.62-23.21)	6.52 (1.32-22.01)	9.49 (4.93-15.62)	
WBC	$\bar{x}$±SD	11.70±4.322	11.95±6.000	9.63±1.796	0.447
(10^9/L)	Mdn (Min-Max)	10.38 (5.81-18.51)	11.78 (11.67-22.68)	9.55 (7.39-12.98)	
LYM	$\bar{x}$±SD	6.44±2.383	6.02±2.687	5.12±0.944	0.476
(10^9/L)	Mdn (Min-Max)	5.50 (3.20-10.80)	5.80 (0.90-13.40)	5.25 (3.50-6.40)	
%LYM	$\bar{x}$±SD	55.42±5.203	50.67±7.132	53.76±8.476	0.076
(%)	Mdn (Min-Max)	56.19 (45.75-63.68)	50.22 (36.91-64.68)	56.06 (39.06-63.29)	
MONO	$\bar{x}$±SD	1.45±0.652	1.44±0.746	1.12±0.394	0.440
(10^9/L)	Mdn (Min-Max)	1.30 (0.50-2.60)	1.30 (0.30-3.60)	1.10 (0.70-2.00)	
%MONO	$\bar{x}$±SD	12.30±2.813	2.16±3.204	11.54±2.835	0.835
(%)	Mdn (Min-Max)	12.52 (8.59-16.69)	12.78 (7.21-17.96)	11.08 (7.71-15.41)	
GRA	$\bar{x}$±SD	3.83±1.546	4.51±2.102	3.41±1.162	0.218
(10^9/L)	Mdn (Min-Max)	3.60 (1.60-6.90)	4.70 (0.50-9.70)	3.20 (2.10-5.10)	
RBC	$\bar{x}$±SD	4.32±0.349	4.10±0.642	4.19±0.662	0.720
(10^{12}/L)	Mdn (Min-Max)	4.26 (3.71-5.16)	4.12 (2.75-5.31)	4.14 (3.27-5.18)	
HGB	$\bar{x}$±SD	7.71±0.325	7.10±0.956	7.21±0.843	0.086
(g/dL)	Mdn (Min-Max)	7.70 (7.10-8.20)	7.30 (4.80-8.30)	7.25 (5.60-8.40)	
MCV	$\bar{x}$±SD	53.61±6.110	51.20±5.527	51.66±4.561	0.306
(fL)	Mdn (Min-Max)	54.35 (42.80-60.80)	48.80 (45.20-64.60)	50.10 (46.50-59.70)	
HCT	$\bar{x}$±SD	22.69±1.563	20.79±2.955	21.43±2.739	0.063
(%)	Mdn (Min-Max)	23.30 (20.00-24.70)	21.50 (12.50-25.10)	22.05 (16.00-25.20)	
MCH	$\bar{x}$±SD	17.91±1.409	17.41±1.792	17.31±1.226	0.371
(pg)	Mdn (Min-Max)	17.80 (14.90-20.60)	17.30 (14.10-21.80)	17.25 (15.00-19.10)	
MCHC	$\bar{x}$±SD	34.08±2.460	34.23±2.238	33.71±2.103	0.648
(g/dL)	Mdn (Min-Max)	33.40 (30.70-40.00)	33.90 (29.20-38.60)	33.25 (30.90-37.50)	
RDW	$\bar{x}$±SD	20.61±3.094	21.55±2.489	21.23±2.279	0.286
(%)	Mdn (Min-Max)	20.00 (16.90-27.20)	21.70 (13.80-26.00)	21.35 (18.30-24.10)	
PLT	$\bar{x}$±SD	1037.47±484.379	1016.03±398.206	1178.40±430.743	0.575
(10^9/L)	Mdn (Min-Max)	1167.00 (401.00-1838.00)	996.00 (143.00-1837.00)	1176.50 (607.00-1741.00)	
MPV	$\bar{x}$±SD	11.33±0.494	11.30±0.484	11.11±0.657	0.545
(fL)	Mdn (Min-Max)	11.10 (10.60-12.00)	11.20 (10.50-12.30)	10.95 (10.20-12.30)	
LPLT	$\bar{x}$±SD	1.16±0.495	1.13±0.412	1.29±0.423	0.663
(%)	Mdn (Min-Max)	1.29 (0.47-1.95)	1.12 (0.17-1.95)	1.26 (0.73-1.86)	
PDW	$\bar{x}$±SD	8.36±0.745	8.24±0.903	7.89±0.989	0.321
(%)	Mdn (Min-Max)	8.40 (7.00-9.50)	8.00 (6.40-10.30)	7.60 (6.30-9.50)	

$\bar{x}$, mean; SD, standard deviation; Mdn, median; Min-Max, minimum and maximum value.

Table S5. The relationship between *C1R* genotypes and the values of hematological and biochemical indices in older piglets.

Indices		Genotypes			*P*-value
		CC, n=29	TC, n=113	TT, n=57	
TIBC (μmol/L)	$\bar{x}$±SD	97.55±23.496	92.15±23.333	96.24±20.700	0.213
	Mdn (Min-Max)	102.09 (44.42-138.45)	85.52 (20.42-150.27)	98.68 (35.99-134.68)	
Fe (μmol/L)	$\bar{x}$±SD	21.79±5.406	21.86±4.394	22.03±4.774	0.952
	Mdn (Min-Max)	21.49 (9.31-37.79)	21.67 (6.63-34.74)	22.03 (7.70-37.43)	
% Tf (%)	$\bar{x}$±SD	23.31±6.482	25.56±10.734	24.25±8.456	0.300
	Mdn (Min-Max)	23.06 (8.51-39.52)	25.27 (6.75-41.36)	23.96 (6.42-59.20)	
		n=25	n=104	n=52	
%LYM (%)	$\bar{x}$±SD	38.17±5.848	38.89±7.059	39.92±6.626	0.264
	Mdn (Min-Max)	36.82 (25.48-49.45)	38.17 (22.93-57.89)	41.20 (16.36-51.44)	
MONO (10^9/L)	$\bar{x}$±SD	2.05±0.672	1.90±0.619	1.85±0.836	0.170
	Mdn (Min-Max)	2.00 (1.10-3.40)	1.70 (0.90-3.90)	1.65 (0.70-4.60)	
%MONO (%)	$\bar{x}$±SD	9.27±2.835	8.90±2.082	9.59±2.376	0.260
	Mdn (Min-Max)	8.41 (5.17-15.11)	8.42 (5.53-16.67)	8.51 (5.73-16.40)	
%GRA (%)	$\bar{x}$±SD	52.60±7.009	52.29±7.912	50.60±7,647	0.217
	Mdn (Min-Max)	53.76 (35.71-69.42)	53.46 (32.21-69.99)	49.31 (35.07-77.71)	
RBC (10^{12}/L)	$\bar{x}$±SD	6,53±0.469	6.51±0.514	6.39±0.583	0.244
	Mdn (Min-Max)	6.58 (5.55-7.56)	6.54 (4.85-7.89)	6.34 (5.51-7.61)	
HCT (%)	$\bar{x}$±SD	32.32±3.299	33.17±3.905	31.40±3.983	0.054
	Mdn (Min-Max)	32.40 (24.40-37.70)	33.40 (20.60-41.80)	32.50 (23.10-37.70)	
MCHC (g/dL)	$\bar{x}$±SD	34.49±1.885	34.46±2.392	34.67±2.249	0.719
	Mdn (Min-Max)	34.30 (31.20-38.80)	34.20 (28.80-48.00)	34.70 (29.70-40.50)	
RDW (%)	$\bar{x}$±SD	15.86±2.734	16.01±2.899	16.76±2.323	0.254
	Mdn (Min-Max)	15.40 (11.00-20.90)	16.45 (10.60-22.10)	16.75 (11.60-21.20)	
PLT (10^9/L)	$\bar{x}$±SD	1055.24±314.716	975.61±346.537	928.98±351.793	0.561
	Mdn (Min-Max)	1089.00 (600.00-1680.00)	945.00 (52.00-1744.00)	952.50 (55.00-1590.00)	
MPV (fL)	$\bar{x}$±SD	11.04±0.454	10.93±0.674	11.15±0.734	0.086
	Mdn (Min-Max)	11.10 (10.20-12.10)	10.80 (9.50-13.30)	11.10 (9.60-12.60)	
LPLT (%)	$\bar{x}$±SD	1.16±0.324	1.06±0.355	1.03±0.368	0.520
	Mdn (Min-Max)	1.21 (0.68-1.80)	1.04 (0.05-1.83)	1.11 (0.06-1.72)	

$\bar{x}$, mean; SD, standard deviation; Mdn, median; Min-Max, minimum and maximum value.

Table S6. The relationship between *C5* genotypes and the values of hematological and biochemical indices in younger piglets.

Indices		Genotypes AA+AC, n=149	CC, n=125	*P*-value
HDL-ch (mg/dL)	$\bar{x}$±SD	61.52±13.866	62.92±15.697	0.509
	Mdn (Min-Max)	61.00 (30.00-106.00)	62.00 (29.00-112.00)	
TIBC (µmol/L)	$\bar{x}$±SD	125.85±32.241	126.84±30.754	0.618
	Mdn (Min-Max)	123.31 (54.63-216.35)	127.25 (50.33-213.13)	
Fe (µmol/L)	$\bar{x}$±SD	13.44±8.598	13.07±6.917	0.716
	Mdn (Min-Max)	11.10 (2.69-51.22)	10.75 (3.22-41.55)	
% Tf (%)	$\bar{x}$±SD	11.44±7.513	11.28±7.104	0.937
	Mdn (Min-Max)	9.17 (1.32-40.74)	8.87 (1.76-35.13)	
		n=125	n=96	
WBC (10^9/L)	$\bar{x}$±SD	14.92±5.545	15.96±5.192	0.121
	Mdn (Min-Max)	14.17 (1.67-35.89)	15.45 (5.81-30.64)	
LYM (10^9/L)	$\bar{x}$±SD	6.83±2.682	7.36±2.493	0.080
	Mdn (Min-Max)	6.60 (0.90-19.10)	7.40 (3.20-17.20)	
%LYM (%)	$\bar{x}$±SD	46.65±9.004	46.99±8.694	0.899
	Mdn (Min-Max)	46.61 (25.51-64.68)	46.86 (28.30-63.68)	
GRA (10^9/L)	$\bar{x}$±SD	6.64±3.204	6.90±3.060	0.552
	Mdn (Min-Max)	6.00 (0.50-15.70)	6.15 (1.60-15.40)	
%GRA (%)	$\bar{x}$±SD	43.32±10.093	42.36±9.069	0.595
	Mdn (Min-Max)	42.43 (22.39-67.88)	41.66 (24.50-64.08)	
RBC (10^{12}/L)	$\bar{x}$±SD	5.45±1.088	5.50±1.004	0.846
	Mdn (Min-Max)	5.60 (2.75-7.64)	5.69 (2.25-7.41)	
HGB (g/dL)	$\bar{x}$±SD	9.39±2.019	9.32±1.628	0.875
	Mdn (Min-Max)	9.10 (4.80-15.20)	9.40 (3.90-12.60)	
MCV (fL)	$\bar{x}$±SD	50.95±5.353	50.58±5.753	0.432
	Mdn (Min-Max)	50.20 (38.90-62.70)	50.00 (39.70-64.60)	
HCT (%)	$\bar{x}$±SD	27.65±5.933	27.53±5.219	0.903
	Mdn (Min-Max)	27.60 (12.50-43.30)	27.40 (13.40-39.60)	
MCH (pg)	$\bar{x}$±SD	17.20±1.550	17.00±1.514	0.250
	Mdn (Min-Max)	17.30 (13.60-20.60)	16.85 (14.00-21.80)	
MCHC (g/dL)	$\bar{x}$±SD	34.00±2.319	33.99±2.732	0.918
	Mdn (Min-Max)	34.00 (28.20-40.60)	34.05 (28.20-42.70)	
RDW (%)	$\bar{x}$±SD	17.74±3.542	18.18±2.904	0.198
	Mdn (Min-Max)	17.10 (11.10-27.20)	17.85 (11.70-27.80)	
PLT (10^9/L)	$\bar{x}$±SD	965.22±393.122	1020.05±400.815	0.394
	Mdn (Min-Max)	953.00 (55.00-1842.00)	973.00 (152.00-2286.00)	
MPV (fL)	$\bar{x}$±SD	11.04±0.523	11.15±0.503	0.159
	Mdn (Min-Max)	11.00 (9.70-12.40)	11.10 (10.20-12.30)	
LPLT (%)	$\bar{x}$±SD	1.06±0.416	1.13±0.416	0.276
	Mdn (Min-Max)	1.02 (0.06-1.95)	1.08 (0.17-2.40)	
PDW (%)	$\bar{x}$±SD	7.68±1.096	7.86±0.915	0.143
	Mdn (Min-Max)	7.60 (5.50-12.20)	7.95 (5.90-9.50)	

$\bar{x}$, mean; SD, standard deviation; Mdn, median; Min-Max, minimum and maximum value.

Table S7. The relationship between *C5* genotypes and the values of hematological and biochemical indices in healthy piglets.

Indices		Genotypes AA+AC, n=73	CC, n=60	*P*-value
HDL-ch	$\bar{x}$±SD	60.79±13.743	64.73±13.411	0.171
(mg/dL)	Mdn (Min-Max)	60.00 (30.00-97.00)	62.00 (36.00-106.00)	
TIBC	$\bar{x}$±SD	122.83±30.990	127.02±26.274	0.268
(µmol/L)	Mdn (Min-Max)	122.50 (64.48-198.44)	128.24 (72.36-185.73)	
Fe	$\bar{x}$±SD	13.91±8.608	12.97±6.712	0.691
(µmol/L)	Mdn (Min-Max)	11.64 (3.58-51.22)	10.93 (3.58-38.51)	
% Tf	$\bar{x}$±SD	12.31±7.944	11.02±6.778	0.507
(%)	Mdn (Min-Max)	10.41 (2.16-40.74)	9.01 (2.29-35.13)	
WBC	$\bar{x}$±SD	15.21±3.602	15.55±3.254	0.500
(10^9/L)	Mdn (Min-Max)	14.67 (6.51-21.54)	15.39 (8.56-22.85)	
LYM	$\bar{x}$±SD	6.86±2.001	7.17±1.747	0.348
(10^9/L)	Mdn (Min-Max)	6.80 (3.40-12.60)	7.25 (3.80-12.90)	
%LYM	$\bar{x}$±SD	45.11±7.541	46.49±8.196	0.312
(%)	Mdn (Min-Max)	45.05 (28.22-60.85)	46.00 (28.30-62.88)	
MONO	$\bar{x}$±SD	1.46±0.495	1.60±0.560	0.196
(10^9/L)	Mdn (Min-Max)	1.40 (0.60-3.00)	1.50 (0.60-2.70)	
%MONO	$\bar{x}$±SD	9.66±2.505	10.20±2.585	0.188
(%)	Mdn (Min-Max)	8.94 (6.50-16.08)	9.15 (6.22-16.97)	
GRA	$\bar{x}$±SD	6.88±2.100	6.79±2.159	0.661
(10^9/L)	Mdn (Min-Max)	6.50 (2.40-11.60)	6.35 (2.60-12.70)	
%GRA	$\bar{x}$±SD	45.18±8.539	43.40±8.426	0.215
(%)	Mdn (Min-Max)	44.70 (24.59-64.73)	43.08 (28.18-62.61)	
RBC	$\bar{x}$±SD	5.93±0.661	5.91±0.617	0.876
(10^{12}/L)	Mdn (Min-Max)	5.92 (4.53-7.64)	5.87 (4.27-7.18)	
HGB	$\bar{x}$±SD	10.15±1.530	9.94±1.108	0.632
(g/dL)	Mdn (Min-Max)	10.00 (8.00-15.20)	9.80 (8.20-12.60)	
MCV	$\bar{x}$±SD	50.64±5.424	50.31±5.648	0.706
(fL)	Mdn (Min-Max)	49.80 (40.20-61.30)	50.00 (39.70-62.10)	
HCT	$\bar{x}$±SD	29.99±4.519	29.51±3.839	0.823
(%)	Mdn (Min-Max)	29.70 (21.40-43.30)	29.95 (20.10-39.60)	
MCH	$\bar{x}$±SD	17.05±1.560	16.82±1.388	0.363
(pg)	Mdn (Min-Max)	17.20 (13.60-20.60)	16.70 (14.00-20.10)	
MCHC	$\bar{x}$±SD	33.91±2.482	33.83±2.950	0.873
(g/dL)	Mdn (Min-Max)	34.10 (28.20-40.60)	34.00 (28.20-42.70)	
RDW	$\bar{x}$±SD	16.45±2.608	17.03±2.032	0.074
(%)	Mdn (Min-Max)	16.20 (11.10-23.50)	16.90 (11.70-21.90)	
PLT	$\bar{x}$±SD	982.79±373.104	1015.25±350.825	0.529
(10^9/L)	Mdn (Min-Max)	957.00 (211.00-1842.00)	996.00 (327.00-1660.00)	
MPV	$\bar{x}$±SD	10.92±0.522	11,08±0.484	0.066
(fL)	Mdn (Min-Max)	10.90 (9.70-12.40)	11.10 (10.20-12.20)	
LPLT	$\bar{x}$±SD	1.07±0.394	1,12±0.368	0.345
(%)	Mdn (Min-Max)	1.02 (0.23-1.95)	1.12 (0.37-1.79)	
PDW	$\bar{x}$±SD	7.42±0.994	7.68±0.846	0.089
(%)	Mdn (Min-Max)	7.30 (5.50-9.60)	7.55 (5.90-9.40)	

$\bar{x}$, mean; SD, standard deviation; Mdn, median; Min-Max, minimum and maximum value.

Table S8. The relationship between *C5* genotypes and the values of hematological and biochemical indices in piglets deviated from physiological norm.

Indices		Genotypes<br>AC, n=15	 CC, n=13	*P*-value
HDL-ch	$\bar{x}$±SD	55.34±8.853	55.69±19.098	0.661
(mg/dL)	Mdn (Min-Max)	55.00 (37.00-71.00)	54.00 (31.00-86.00)	
TIBC	$\bar{x}$±SD	133.94±28.676	120.13±26.51	0.249
(µmol/L)	Mdn (Min-Max)	135.40 (88.83-186.26)	113.91 (87.94-165.49)	
Fe	$\bar{x}$±SD	19.13±11.166	13.96±6.597	0.289
(µmol/L)	Mdn (Min-Max)	15.94 (5.37-38.87)	10.75 (5.19-28.30)	
% Tf	$\bar{x}$±SD	15.97±10.635	13.11±8.527	0.612
(%)	Mdn (Min-Max)	13.24 (3.09-32.28)	9.28 (3.22-31.54)	
WBC	$\bar{x}$±SD	23.34±6.109	24.18±4.728	0.713
(10^9/L)	Mdn (Min-Max)	24.33 (10.18-35.89)	25.17 (12.17-30.64)	
LYM	$\bar{x}$±SD	9.23±4.279	10.27±3.541	0.580
(10^9/L)	Mdn (Min-Max)	10.70 (2.70-19.10)	9.00 (5.40-17.20)	
%LYM	$\bar{x}$±SD	37.87±10.192	24.28±9.90	0.182
(%)	Mdn (Min-Max)	39.70 (25.50-53.22)	41.68 (29.21-56.14)	
MONO	$\bar{x}$±SD	1.95±0.655	2.37±0.867	0.133
(10^9/L)	Mdn (Min-Max)	2.00 (0.70-3.50)	2.20 (1.10-4.50)	
GRA	$\bar{x}$±SD	12.18±2.702	11.57±3.082	0.549
(10^9/L)	Mdn (Min-Max)	12.20 (6.70-15.70)	12.90 (5.70-15.40)	
%GRA	$\bar{x}$±SD	53.95±10.70	48.12±10.346	0.141
(%)	Mdn (Min-Max)	51.10 (37.06-67.87)	48.88 (34.88-64.08)	
RBC	$\bar{x}$±SD	6.40±0.591	5.83±1.299	0.222
(10^{12}/L)	Mdn (Min-Max)	6.54 (5.36-7.59)	5.99 (2.25-7.41)	
HGB	$\bar{x}$±SD	11.02±1.835	9.90±2.142	0.153
(g/dL)	Mdn (Min-Max)	11.40 (8.10-14.30)	10.70 (3.90-12.20)	
MCV	$\bar{x}$±SD	49.35±5.461	50.77±5.551	0.629
(fL)	Mdn (Min-Max)	50.60 (38.90-57.00)	50.50 (40.90-59.60)	
HCT	$\bar{x}$±SD	31.73±5.629	29.23±6.308	0.279
(%)	Mdn (Min-Max)	33.50 (20.80-41.60)	30.10 (13.40-37.40)	
MCH	$\bar{x}$±SD	17.07±1.655	17.00±1.329	0.836
(pg)	Mdn (Min-Max)	17.40 (14.10-19.30)	16.80 (15.30-19.20)	
MCHC	$\bar{x}$±SD	34.81±1.934	33.75±2.162	0.197
(g/dL)	Mdn (Min-Max)	35.20 (31.80-39.40)	34.10 (29.10-37.60)	
PLT	$\bar{x}$±SD	699.80±374.512	962.69±521.829	0.153
(10^9/L)	Mdn (Min-Max)	666.00 (55.00-1408.00)	935.00 (152.00-2286.00)	
MPV	$\bar{x}$±SD	11.10±0.393	11.17±0.505	0.548
(fL)	Mdn (Min-Max)	11.20 (10.20-11.70)	11.30 (10.30-12.00)	
LPLT	$\bar{x}$±SD	0.78±0.424	1.06±0.544	0.107
(%)	Mdn (Min-Max)	0.76 (0.06-1.59)	1.00 (0.17-2.40)	
PDW	$\bar{x}$±SD	7.84±1.581	7.81±1.119	0.661
(%)	Mdn (Min-Max)	8.10 (5.50-12.20)	8.00 (6.20-9.30)	

$\bar{x}$, mean; SD, standard deviation; Mdn, median; Min-Max, minimum and maximum value.

Table S9. The relationship between *C5* genotypes and the values of hematological and biochemical indices in anemic piglets.

Indices		Genotypes AA+AC, n=37	Genotypes CC, n=23	*P*-value
HDL-ch (mg/dL)	$\bar{x}$±SD<br>Mdn (Min-Max)	66.35±16.618<br>65.00 (41.00-106.00)	58.61±16.256<br>61.00 (29.00-96.00)	0.107
TIBC (μmol/L)	$\bar{x}$±SD<br>Mdn (Min-Max)	129.12±32.827<br>130.03 (54.63-208.83)	137.55±39.788<br>138.62 (50.33-205.61)	0.382
Fe (μmol/L)	$\bar{x}$±SD<br>Mdn (Min-Max)	11.19±5.336<br>10.75 (2.69-25.43)	11.01±5.950<br>9.13 (3.22-24.72)	0.605
% Tf (%)	$\bar{x}$±SD<br>Mdn (Min-Max)	9.20±4.545<br>8.58 (1.32-19.24)	8.89±5.927<br>6.45 (1.76-23.21)	0.354
WBC (10^9/L)	$\bar{x}$±SD<br>Mdn (Min-Max)	10.95±4.420<br>10.49 (1.67-22.68)	12.39±4.694<br>12.07 (5.81-22.45)	0.274
LYM (10^9/L)	$\bar{x}$±SD<br>Mdn (Min-Max)	5.81±2.487<br>5.50 (0.90-13.40)	6.24±2.319<br>5.90 (3.20-10.40)	0.503
%LYM (%)	$\bar{x}$±SD<br>Mdn (Min-Max)	53.26±6.585<br>53.42 (38.86-64.68)	50.95±7.914<br>50.21 (36.91-63.68)	0.213
MONO (10^9/L)	$\bar{x}$±SD<br>Mdn (Min-Max)	1.24±5.532<br>1.10 (0.30-2.30)	1.63±0.818<br>1.30 (0.50-3.60)	0.077
%MONO (%)	$\bar{x}$±SD<br>Mdn (Min-Max)	11.67±3.075<br>12.44 (7.21-17.96)	12.77±2.846<br>13.84 (8.28-16.63)	0.125
GRA (10^9/L)	$\bar{x}$±SD<br>Mdn (Min-Max)	3.92±1.785<br>3.80 (0.50-8.70)	4.54±1.993<br>4.30 (1.60-9.70)	0.369
%GRA (%)	$\bar{x}$±SD<br>Mdn (Min-Max)	35.33±6.383<br>33.79 (22.39-53.54)	36.39±6.822<br>37.54 (24.50-49.60)	0.558
RBC (10^{12}/L)	$\bar{x}$±SD<br>Mdn (Min-Max)	4.12±0.642<br>4.11 (2.75-5.31)	4.24±0.484<br>4.28 (3.27-5.16)	0.621
HGB (g/dL)	$\bar{x}$±SD<br>Mdn (Min-Max)	7.21±0.941<br>7.40 (4.80-8.30)	7.38±0.695<br>7.60 (5.70-8.40)	0.715
MCV (fL)	$\bar{x}$±SD<br>Mdn (Min-Max)	52.21±5.035<br>51.00 (45.20-62.70)	51.24±6.338<br>48.05 (42.80-64.60)	0.294
HCT (%)	$\bar{x}$±SD<br>Mdn (Min-Max)	21.37±3.077<br>22.10 (12.50-25.20)	21.38±2.088<br>22.10 (16.80-24.70)	0.654
MCH (pg)	$\bar{x}$±SD<br>Mdn (Min-Max)	17.54±1.472<br>17.50 (14.50-20.60)	17.48±1.854<br>17.30 (14.10-21.80)	0.825
MCHC (g/dL)	$\bar{x}$±SD<br>Mdn (Min-Max)	33.83±2.105<br>33.40 (30.60-38.60)	34.55±2.431<br>34.30 (29.20-40.00)	0.218
RDW (%)	$\bar{x}$±SD<br>Mdn (Min-Max)	21.35±2.708<br>21.70 (13.80-27.20)	21.11±2.484<br>20.90 (17.50-26.00)	0.637
PLT (10^9/L)	$\bar{x}$±SD<br>Mdn (Min-Max)	1038.16±404.866<br>1012.00 (143.00-1837.00)	1065.00±459.355<br>1006.00 (471.00-1838.00)	0.964
MPV (fL)	$\bar{x}$±SD<br>Mdn (Min-Max)	11.25±0.511<br>11.30 (10.20-12.20)	11.31±0.529<br>11.10 (10.60-12.30)	0.789
LPLT (%)	$\bar{x}$±SD<br>Mdn (Min-Max)	1.15±0.415<br>1.16 (0.17-1.95)	1.19±0.467<br>1.12 (0.56-1.95)	0.988
PDW (%)	$\bar{x}$±SD<br>Mdn (Min-Max)	8.12±0.916<br>7.90 (6.30-10.30)	8.36±0.817<br>8.30 (7.00-9.50)	0.247

$\bar{x}$, mean; SD, standard deviation; Mdn, median; Min-Max, minimum and maximum value.

Table S10. The relationship between *C5* genotypes and the values of hematological and biochemical indices in older piglets.

Indices		Genotypes AC, n=76	CC, n=123	*P*-value
HDL-ch (mg/dL)	$\bar{x}$±SD	50.54±10.633	50.14±12.515	0.556
	Mdn (Min-Max)	50.00 (29.00-79.00)	47.00 (20.00-83.00)	
Fe (μmol/L)	$\bar{x}$±SD	22.06±4.482	21.80±4.749	0.838
	Mdn (Min-Max)	21.58 (9.31-37.79)	22.03 (6.63-37.43)	
		n=69	n=112	
LYM (10^9/L)	$\bar{x}$±SD	7.69±1.984	8.32±2.488	0.119
	Mdn (Min-Max)	7.60 (3.70-12.80)	7.95 (4.00-16.10)	
%LYM (%)	$\bar{x}$±SD	40.44±6.126	38.26±7.038	0.055
	Mdn (Min-Max)	41.18 (26.47-57.89)	38.27 (16.36-53.98)	
%MONO (%)	$\bar{x}$±SD	9.05±2.128	9.22±2.391	0.855
	Mdn (Min-Max)	8.43 (6.33-16.67)	8.45 (5.17-16.40)	
%GRA (%)	$\bar{x}$±SD	50.59±6.883	52.62±8.125	0.126
	Mdn (Min-Max)	49.79 (32.22-65.58)	52.97 (35.07-77.71)	
RBC (10^{12}/L)	$\bar{x}$±SD	6.54±0.466	6.44±0.542	0.260
	Mdn (Min-Max)	6.59 (5.53-7.89)	6.48 (4.85-7.83)	
HGB (g/dL)	$\bar{x}$±SD	11.43±1.039	11.05±1.071	0.072
	Mdn (Min-Max)	11.30 (9.20-14.10)	11.20 (8.10-13.20)	
MCV (fL)	$\bar{x}$±SD	50.49±3.920	50.13±4.252	0.759
	Mdn (Min-Max)	50.50 (40.90-58.60)	50.30 (41.00-59.70)	
HCT (%)	$\bar{x}$±SD	32.97±3.574	32.28±4.097	0.587
	Mdn (Min-Max)	33.00 (23.00-41.80)	33.15 (20.60-39.70)	
MCHC (g/dL)	$\bar{x}$±SD	34.70±1.654	34.41±2.589	0.155
	Mdn (Min-Max)	34.60 (29.70-40.00)	34.20 (28.80-48.00)	
RDW (%)	$\bar{x}$±SD	16.11±2.668	16.26±2.782	0.674
	Mdn (Min-Max)	16.30 (11.30-21.10)	16.80 (10.60-22.10)	
PLT (10^9/L)	$\bar{x}$±SD	972.64±356.634	973.56±337.98	0.808
	Mdn (Min-Max)	935.00 (109.00-1744.00)	999.00 (52.00-1645.00)	
MPV (fL)	$\bar{x}$±SD	11.08±0.688	10.97±0.659	0.492
	Mdn (Min-Max)	11.00 (9.90-13.30)	10.90 (9.50-12.60)	
LPLT (%)	$\bar{x}$±SD	1.07±0.366	1.06±0.349	0.776
	Mdn (Min-Max)	1.04 (0.11-1.83)	1.10 (0.05-1.74)	
PDW (%)	$\bar{x}$±SD	7.59±1.009	7.47±1.053	0.471
	Mdn (Min-Max)	7.60 (5.50-9.60)	7.20 (5.20-9.90)	

$\bar{x}$, mean; SD, standard deviation; Mdn, median; Min-Max, minimum and maximum value.
